# Eigenvector Centrality is a Metric of Elastomer Modulus, Heterogeneity, and Damage

**DOI:** 10.1038/s41598-017-00818-0

**Published:** 2017-04-27

**Authors:** P. M. Welch, C. F. Welch

**Affiliations:** 1 Theoretical Division, Los Alamos National Laboratory, Los Alamos, New Mexico, 87544 USA; 2 Materials Science and Technology Division, Los Alamos National Laboratory, Los Alamos, New Mexico, 87544 USA

## Abstract

We present an application of eigenvector centrality to encode the connectivity of polymer networks resolved at the micro- and meso-scopic length scales. This method captures the relative importance of different nodes within the network structure and provides a route toward the development of a statistical mechanics model that correlates connectivity with mechanical response. This scheme may be informed by analytical and semi-analytical models for the network structure, or through direct experimental examination. It may be used to predict the reduction in mechanical performance for heterogeneous materials subjected to specific modes of damage. Here, we develop the method and demonstrate that it leads to the prediction of established trends in elastomers. We also apply the model to the case of a self-healing polymer network reported in the literature, extracting insight about the fraction of bonds broken and re-formed during strain and recovery.

## Introduction

Predicting the macroscopic mechanical response of polymeric solids based on the underlying material structure remains an elusive problem, even after many decades have passed since the development of the earliest molecularly-aware theories. Rather than applying a detailed evaluation of structure at either the micro- or meso-scopic length scales, in practice, one often resorts to using toy mechanical models that require many empirical coefficients and are disconnected from the underlying molecular picture. Though much effort has been invested in capturing the rich phenomena at the molecular scale^1–3^, including chain conformational statistics, chemical and physical cross-linking, and entanglements, we still lack a satisfactory means to pass that information up to the macroscopic length scales^4^. This problem becomes more challenging when damage occurs during the use of the material and is especially relevant to the emerging field of self-healing materials^5–7^, in which the mechanical characteristics evolve with time as the result of molecular processes.

Here, we propose that developments in graph theory that are finding broad application in the arena of information science^8–10^ may provide a systematic means to encode specific structural details and to do so in a manner cognizant of the relative importance of different portions of the material. This latter trait should prove especially useful in evaluating the robustness of a given material in a specific application. Moreover, these developments also provide a means to capture the network details in a reduced order model.

Eigenvector centrality^11^ and its variants find use in such diverse information science applications as contemporary search engines^8^ and community identification^9, 10^. It is especially useful in ranking the importance of each node in a network based not only on its own functionality or degree, but also on the connectivity of its neighbors. To gather this information, one need only find the principal eigenvector of the adjacency matrix for the network. That is, one finds the eigenvector with the largest eigenvalue for the matrix that has non-zero element entries for pairs of nodes that are connected (when represented in the natural basis of node-identity). The relative value of the elements of the eigenvector indicates the relative importance that the corresponding node holds in the network. In the context of academic papers, this may highlight those articles that hold the most impact in a given scientific community. In the context of polymers, we postulate that network importance ranking can inform models of mechanical response. While the application of graph theory ideas to polymer mechanics has a long history^12^, the application of centrality appears not to have been previously explored.

The adjacency matrix $\hat{A}$ provides a table denoting which nodes in the network are linked. For example, $\hat{A}$ for a linear chain whose nodes are numbered consecutively with node identification number *n* ranging from 1 to N is given by ${A}_{i,j}=\delta (j-(i+1))+\delta (j-(i-1))$ when using the node identification as the basis. The principal eigenvector ${\mathop{e}\limits^{\longrightarrow}}_{1}$ holds the importance ranking of each node within the network. It has unit magnitude, but its elements do not sum to one. The eigenvalue *λ* is the maximum of either the importance-weighted average degree of the network or the square root of the largest value of degree found within the network^13^. Thus, it is a measure of the functionality of the most important nodes and encodes the relative quantities of different kinds of bonds in the network.

We construct a simple statistical mechanics model to exploit these characteristics via the following *ansatz*. Let us define an importance operator $\hat{\iota }$ that is represented in the $\mathop{n}\limits^{\longrightarrow}$ basis as a diagonal matrix with elements that are simply those of ${\mathop{e}\limits^{\longrightarrow}}_{1}$. Since the elements of ${\mathop{e}\limits^{\longrightarrow}}_{1}$ provide the relative importance of each node in the network, we associate ${\mathop{e}\limits^{\longrightarrow}}_{1}$ with a distribution function that partitions importance within the graph. Note that importance means the relative contribution that a node makes to the network in the sense of its connectedness to other nodes; in short, high importance means a node is connected to many other nodes that are also highly connected. Thus, the trace of $\hat{\iota }$ may be associated with a partition sum $Z\equiv Tr[\hat{\iota }]$. We also identify an underlying Hamiltonian operator $\hat{H}$ such that $Z=Tr[\exp (-{\rm{\Gamma }}\hat{H})]$. Here, ${\rm{\Gamma }}$ is the inverse of the Lagrange multiplier that enforces average degree in the network and is the analog of the inverse thermal energy. We propose that this multiplier is proportional to the probability for any two nodes to form a link, *P*. Within this framework, we define a network free energy $F\equiv -P\,\mathrm{log}(Z)$.

**Figure 1:**
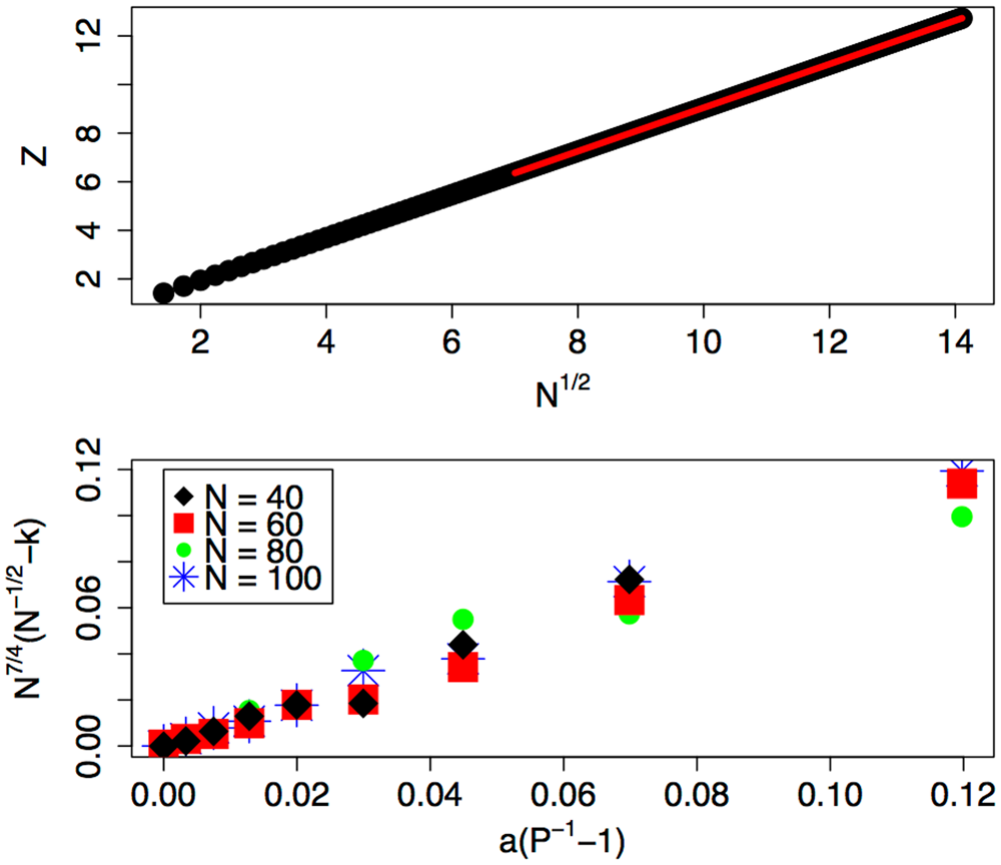
The top plot contains the partition function *Z* as a function of the square root of the number of nodes in a linear graph as calculated by direct evaluation of all of the eigenvectors and eigenvalues. The black circles represent the calculated values, while the red curve is a fit illustrating the proposed *N* ^1/2^ dependence. The bottom plot is the ensemble average value of the elements of the principal eigenvector for random networks as calculated with the power iteration method cast in a form of the proposed model that collapses the data, capturing variation in both *P* and *N*. The data derives from random networks with values of *P* spanning 0.2 to 1.0 and values of *N* ranging from 40 to 100.

Linear chains provide insight into how to link the Young’s tensile modulus *E* to this free energy. Numerical interrogation of a number of linear graphs reveals that the partition sum is well approximated by $Z\approx \frac{2}{3}\xi \frac{{N}^{2}-1}{N}$. We also find that the value of the scale parameter may be approximated by *ξ* ≈ 1.48 × 10^−3^ + 1.39 *N* ^−1/2^ over the range tested herein. If the exponent of −1/2 varies at higher values of *N*, the prefactor merely changes in the following analysis leading to Eq. 1. Recall that *P* is the ratio of the number of bonds formed in the network to the number possible. In the linear case, we have *P* = 2(*N* − 1)/(*N* ^2^ − *N*). Thus, in the large *N* limit, *Z* ∝ *N* ^0.5^, *P* ∝ 1/*N*, and *F* = −*P* log(*Z*) decays as log(*N*)/*N*. See Fig. 1 (top), which contains a plot of *Z* versus *N* ^1/2^ for the linear chain case. Now, for linear chains the modulus *E* drops as 1/*N* ^14^. We therefore predict that the modulus is related to the network free energy by Eq. 1, the guiding hypothesis for our analysis.

1 $$E\propto -F/\mathrm{log}(N)$$

Random networks (Erdös - Renyi graphs^15^) provide a more realistic model for real polymeric solids and have been applied in the past to describe both the onset of a percolated structure^16, 17^, as well as to capture the thermodynamics of elastomers^12^. Consider a set of *N* junctions that form links with any other junction with probability *P*. Here, one does not expect that the node identification number plays a role. Rather, ${e}_{1}(n)\equiv k$, a fixed constant with an average value that varies with *P* and *N*. From numerical interrogation of networks composed of *N* = 40 − 100 junctions and *P* = 0.2 − 1.0, we find that $k=-a{N}^{-\mathrm{7/4}}({P}^{-1}-1)+{N}^{-\mathrm{1/2}}$. See Fig. 1 (bottom), which contains a plot of *N* ^7/4^(*N* ^−1/2^ − *k*) versus *a*(*P* ^−1^ − 1). Here, *a* is a numerical constant approximated by *a* ≈ 0.03. The partition sum then becomes $Z=Nk={N}^{\mathrm{1/2}}-a{N}^{-\mathrm{3/4}}({P}^{-1}-1)$.

We assume that the junction density is fixed and that the volume of the solid grows as *N*. Applying the relationship between *E* and *F* above, Eq. 1, yields: $E\propto P\,\mathrm{log}({N}^{\mathrm{1/2}}-a{N}^{-\mathrm{3/4}}({P}^{-1}-1))/\mathrm{log}(N)$. Since *aN* ^−3/4^(*P* ^−1^ − 1) is always small, one may further simplify the prediction for *E* by expanding the logarithm of *Z* to find that $E\propto P/2-a(1-P)/({N}^{\mathrm{5/4}}\,\mathrm{log}(N))$. Thus, the model predicts an *N* (or size) dependence for *E* only for very small networks, as expected.

The connection to classical rubber elasticity theory may be made clear by making explicit the connection to cross-linking density. In the classical theory, the modulus varies directly with the number of cross-links per unit volume, *X*. This may be approximated as *X* ∝ *PN*/*V* ∝ *PN*/*N*, where *V* is the sample volume. Thus, we find that *E* ∝ *P* ∝ *X*. That is, for a fixed network density, the application of eigenvector centrality and the hypothesis that Eq. 1 holds predicts that the modulus varies directly with cross-link density, in accord with classical models^3^.

**Figure 2:**
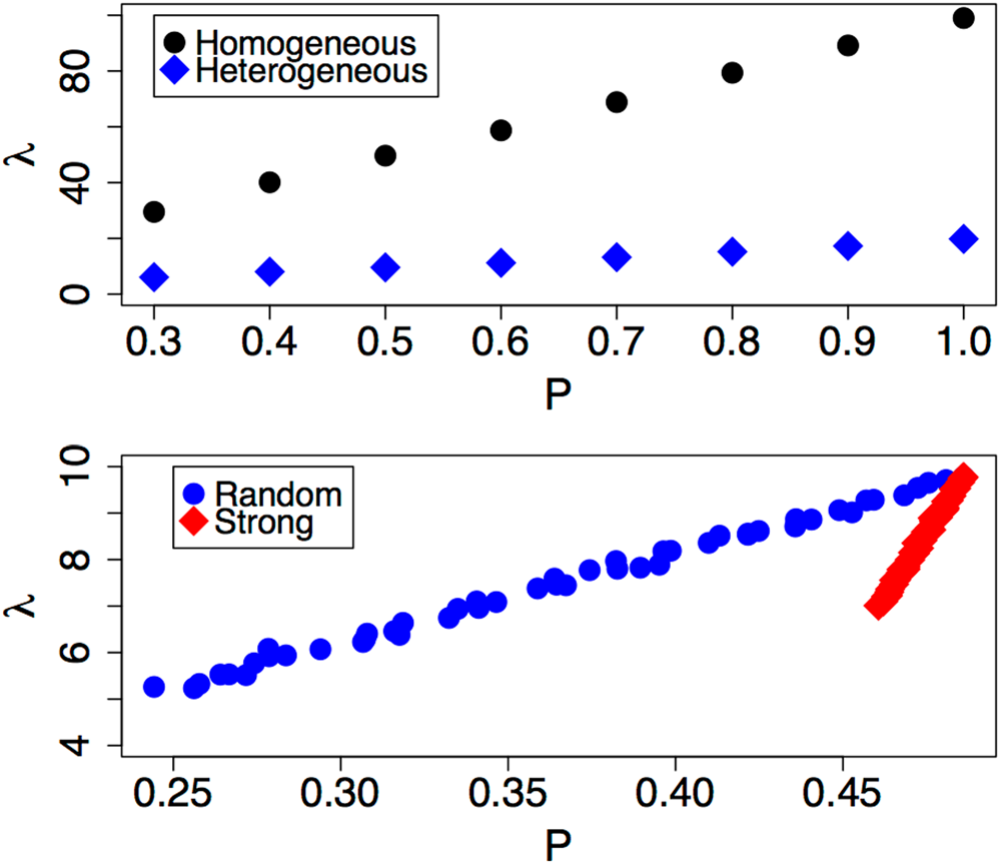
The top plot contains the principal eigenvalue *λ* as a function of bonding probability *P* for example homogeneous and heterogeneous networks. The bottom plot contains *λ* for a heterogenous network under two different modes of link deletion as a function of *P*. Initially, the network is composed of a ratio of 9-to-1 links with weights 0.1 and 1.0. At construction, *P* = 0.5. The mode labeled “Random” involves the random selection of links for deletion. The mode labeled “Strong” corresponds to deleting only links with weight equal to 1.0.

To illustrate how heterogeneity impacts the modulus and how to encode it in the proposed scheme, consider a modification to the random network constructed as above. Let there be a ninety percent chance that a given link is “weak” in the sense that it contributes less to the network structure, while the remaining ten percent are “strong”. This may mimic, for example, a network composed of physical cross-links (weak) and covalent cross-links (strong). We encode this weakness in $\hat{A}$ by assigning a value of 0.1 rather than 1.0 to the entries for the weaker edges. Numerically estimating the modulus *E* confirms that this quantity is linear in *P* for both cases. However, both the homogeneous and heterogeneous networks produce trends that fall nearly upon one another; *F* only provides insight into the global organization of the network (i.e. random versus ordered), but does not probe details at the micro-structural level. The eigenvalue *λ*, presented in Fig. 2 (top), does capture the variations due to heterogeneity; it captures microstructure. Now, *λ* is a measure of the importance-averaged degree in the network and is, thus, proportional to *PN*. Therefore, this formalism provides a direct means to pass specific mesoscale structure up to the macro-scale.

Materials often experience changes in their mechanical properties during their lifecycle^18, 19^. Changes such as mechanical chain scission^6^, chemical degradation^20, 21^, and morphological rearrangement^2^ that result in the loss of macroscopic material properties are often grouped under the rubric of “damage”. Quantifying and predicting these changes based on the micro- and meso-scopic pictures presents a serious challenge when modeling specific materials. Though an active area of research reported in the literature^22^, we still lack a robust mathematical definition for damage that goes beyond noting changes at the macroscopic length scale. Here, we propose such a metric and illustrate its application.

Defining a new measure of damage follows from a straightforward extension of the two observations above: i) *F* captures information about how the network was prepared (random, ordered, etc.) and provides a link to *E* and ii) *λ* captures the micro-structural details and plays a role in the prefactor, discussed below. For a homogenous network, one may simply define a rate of damage as the change in *F* with change in *P*. This will be characteristic of the particular network type and will predict variations in *E* in the homogeneous case. This, however, fails to capture the micro-structural details that play a role in damage in real, heterogenous materials. Rather, we define two damage rates, one that captures network class and one that captures microstructure. Though *F* is insensitive to the particular details of how *P* is changed, *λ* does vary with *P* in a manner that depends upon the specific mode of link deletion employed. For example, consider a heterogeneous network like the one described above, beginning with *P* = 0.5. Figure 2 (bottom) presents two scenarios, the case in which the deletions are carried out randomly and the case in which deletions are performed only on the “strong” links. The latter scenario may correspond to, for example, the case of chemical degredation of the covalent bonds. Clearly, *λ* falls much more quickly as a function of *P* in the case where only the strongly contributing bonds are removed.

We therefore propose a new damage model. First, we introduce a function Ψ to complete the equality between *E* and *F*. It is dependent on *λ*/(*PN*) − Λ in order to preserve the scaling relied upon to arrive at Eq. 1 and a numerical prefactor that absorbs the *log*(*N*) term (discussed further below in the context of the model’s application). We further assume that Ψ is a power law, giving Eq. 2 as our final model form.

2 $$E\equiv {\rm{\Psi }}F=exp(b){(\frac{\lambda }{PN}-{\rm{\Lambda }})}^{m}F$$

The new parameter Λ is the value of *λ*/*PN* upon the formation of a percolated network, while the prefactor *exp*(*b*) and exponent *m* are free fitting parameters that capture the chemical details. Next, we define a global damage rate defined as $\delta F\equiv \frac{dF}{dP}$ and a microstructure damage rate $\delta \lambda \equiv {\frac{d\lambda }{dP}|}_{M}$. Here, the suffix “M” implies that the links are deleted in a specific fashion or mode (e. g. randomly chosen, only those of a certain class, etc.). Combining these two terms yields a total damage rate measure suitable for predicting the trend in *E* under a given mode of link removal:

3 $$\delta E\equiv ({F\frac{d{\rm{\Psi }}}{d\lambda }|}_{M}\delta \lambda +{\rm{\Psi }}\delta F)$$

Clearly, if Eq. 3 does not vary with *λ* under the mode of link deletion, then the variation in *E* resides entirely in the second term. One expects that this is true when bond deletion occurs randomly rather than targeting specific link categories. For example, the slope for the heterogenous case in Fig. 2 (top) is identical to that for the random case in Fig. 2 (bottom) in which all bonds are randomly deleted; one does not expect that the prefactor changes as a function of cross-linking if the relative proportion of the junctions, weak to strong, remains fixed. That is, there is a variation in Ψ only if the ratio of *λ*/(*PN*) changes. The random deletion case, in which *λ*/(*PN*) remains fixed is illustrated by the “Random” line for one specific ratio of “strong” to “weak” in Fig. 2. The “Strong” line corresponds to how *λ* varies with *P* under a non-fixed ratio of linkage types. These slopes correspond to variations of two different functions; that found in the prefactor Ψ and that in *δλ*, respectively.

The recent study by Bao and coworkers^23^ provides an excellent test application for this model, since their polymer structure is well defined, the cross-linking density is controlled, damage is induced, and the nature of the entanglements are well understood for their chemistry. Those investigators prepared a self-healing elastomer composed of chain-extended poly(dimethyl siloxane) (PDMS) linked by moieties (2,6-pyridinedicarboxamide, or H2pdca) that act as ligands in iron complexes. Each of their chain-extended threads had roughly nineteen iron binding sites and eighteen runs of 6,000 g/mol PDMS. To these threads, they added FeCl_3_, which undergoes ligand exchange to form bonds with the polymer threads (via the H2pdca sites) to form a cross-linked network. They demonstrated that the elastomer thus prepared suffered damage upon elongation followed by subsequent healing, supporting the idea that the polymer-iron-polymer bonds were broken and re-formed. The cross-linking density was varied by changing the ratio of the iron-binding sites to the amount of FeCl_3_ added to the material.

The essential step in applying the proposed model to this material lies in generating an ensemble of random adjacency matrices that conform to the known facts about the material. We build this ensemble based on a coarse-grained model of the polymer threads: each thread is composed of nineteen iron-binding sites and eighteen potential PDMS entanglement sites, with the two site types alternating along the chain backbone. If we assume a mono-disperse collection of threads, then we know that there are well-defined static covalent bonds in the system. We give those bonds a weighting of 1 in every realization of $\hat{A}$. We also know that there should be on average nine entanglement sites per thread, since the entanglement molecular weight of PDMS is roughly 12,000 g/mol^24^ and the total molecular weight of the threads is about 108,000 g/mol. Thus, each entanglement site forms a bond with another such site with probability of a half. Those bonds are assigned a weight of 0.1 in $\hat{A}$, reflecting the physical intuition that entanglement plays a lesser role in determining the modulus of the system. More on this choice follows below. Finally, we also have some knowledge of the probability for forming a polymer-iron-polymer bond for a given stoichiometric ratio of ligand to iron. Roughly speaking, each thread must have about two polymer-iron-polymer bonding sites at the onset of a percolated network. This is rough since some chains may be part of the network but form dangling ends, while other chains that satisfy this criteria may be bound only intramolecularly. Given this, examining Bao’s data, we estimate that the FeCl_3_ is only 50% efficient in forming cross-links, since one would need $\tfrac{1}{2}\tfrac{2}{19}\approx \mathrm{5 \% }$ FeCl_3_ upon network formation, while extrapolating the data to zero modulus indicates that the threshold value is closer to 10%. The factor of a half reflects the fact that each polymer-iron-polymer bond has one iron per every two bound polymer sites. That the efficiency is less than one comports with the physical expectation that not every iron finds two polymer ligand sites due to kinetic frustration. Thus, each iron-binding site has a probability *P*_*f*_ ≈ 17% of forming a polymer-iron-polymer bond for the scenario in which one FeCl_3_ molecule is added for every six iron-binding sites (i.e., 1:6 Fe:Hpdca-PDMS), the lowest ratio reported by Bao. To those sites, we assigned a weight of 0.99 in $\hat{A}$. We choose a representative sample size of sixty chains to generate an ensemble of one thousand independent realizations of $\hat{A}$ within this picture, generating average values for *F* and *λ*/(*PN*). This choice, as well as the choices for the weights, set the scale for the two fitting parameters reported below; nevertheless, once these choices are made, quantitative predictions regarding the changes in the numbers of bonds may be calculated. The assignment of the weights for more general problems merits further study. However, for the particular problem and material that we have studied herein, the weights can be arbitrarily set. This is true because we are breaking only one type of bond (polymer-iron-polymer), and those bonds also set the percolation threshold. A straightforward calculation provided in the Appendix demonstrates that changing the weights only impacts the magnitude of the prefactor fitting term in Ψ.

We note that we make no assumptions regarding affine deformation or spatial heterogeneity. Heterogeneity is captured with the numerical evaluation of the eigenvector and eigenvalues. That is, statistical variations across the network are accounted for if they are encoded within the adjacency matrix. To consider something that is strongly heterogeneous spatially, such as a gradient in cross-linking density across a sample, requires modification of the actual numerical simulations of the networks. This can be done by building correlations into the connectivity of the graph. The basic theoretical model, however, is capable of capturing strong heterogeneity.

The details of the algorithm employed herein to both fit the model and apply it to estimate the unknown values of *P*_*f*_ for damaged samples are given below. Note that three functions are used to invert the model so that *P*_*f*_ may be found for a given value of the modulus and one function is used to extrapolate the value of Λ. These functional forms are motivated by examination of the data rather than any theoretical considerations. The former three are simple functions that fit the numerical data and whose estimates are ultimately checked for consistency in step 6. The latter function is a straightforward linear interpolation. The fit coefficients (*a*, *b*, *c*, and *m*) are given subscripts indicating in which step they are calculated. Table 1 contains a summary of the dependencies and roles of the major parameters of the theory. The algorithm follows.

1. Construct network models for the values of *P*_*f*_ of interest and perform the centrality calculation: a) Randomly assemble 1000 independent adjacency matrixes for a given value of *P*_*f*_, b) use power iteration to estimate the principal eigenvalue *λ* and eigenvector for each realization, c) calculate the partition sum *Z*, and d) estimate 〈*Z*〉 and 〈*λ*〉 where the averages are across the 1000 samples.
2. Estimate the value of Λ, that is, *λ*/(*PN*) at the percolation threshold of *P*_*f*_ = 0.05: construct a linear fit for *λ*/(*PN*) = *m*_2_ *P*_*f*_ + *b*_2_ for the smallest four values of *P*_*f*_ considered and use the fit parameters *m*_2_ and *b*_2_ to calculate Λ.
3. Fit the experimental moduli values for all the non-strained samples (except that corresponding to the highest iron concentration) to the model using the values of 〈*Z*〉 and 〈*λ*〉 as the independent variable: construct a linear fit to $\mathrm{log}(E/\langle F\rangle )={b}_{3}+{m}_{3}\,\mathrm{log}(\langle \lambda \rangle /(PN)-{\rm{\Lambda }})$, where 〈*F*〉 is calculated from 〈*Z*〉.
4. For a sample with unknown *P*_*f*_, construct an estimate of the sample’s *PN*/*λ* (note that this quantity rather than its inverse is modeled as a matter of numerical convenience): a) fit $PN/\lambda ={a}_{4}({b}_{4}+\exp (-{c}_{4}F))$ for the full range of values calculated in step 1 and b) find the value of *F* (and, thus, *PN*/*λ*) that minimizes the squared difference between the target modulus E* and the model, using the fit in step 4.a - that is, minimize ${[{E}^{\ast }-F\exp ({b}_{3}){(\lambda /(PN)-{\rm{\Lambda }})}^{{m}_{3}}]}^{2}$ over *F* with *λ*/(*PN*) dependent upon the value of *F* via the fit in step 4.a.
5. For a sample with unknown *P*_*f*_, estimate this value using the resulting value of *λ*/(*PN*) from step 4: Construct a linear fit across all values simulated in step 1 above for $\mathrm{log}({P}_{f})={m}_{5}\,\mathrm{log}(\lambda /(PN))+{b}_{5}$ and use it to estimate *P*_*f*_ for the unknown.
6. For a sample with unknown *P*_*f*_, validate the estimate from step 5 by following the procedure in step 1 to more accurately estimate the values of 〈*Z*〉 and 〈*λ*〉 for the sample and to place it on the curve with the known samples.

**Table 1:** The relationships between the model input and fitting parameters to the material structure and chemistry, as well as their roles in the presented algorithm.

Parameter	Physical-Chemical Dependencies and Roles
Bond Weights in Adjacency Matrix	arbitrary settings reflecting different bond types
	ultimately sets the scale for model
	input to the centrality calculation
Probabilities for Entanglement and Covalent Bonds	specific chemistry
	chain molecular weight
	input to centrality calculation
*P*_*f*_	crosslinking efficiency
	ratio of FeCl_3_ to ligand
	input to centrality calculation for known samples
	output from model inversion for unknowns
*λ*/(*PN*)	relative fractions of bond types (heterogeneity)
	output from centrality calculation
Λ	percolation limit
	entanglement probability
	primary chain structure
	estimated from centrality calculation
*F*	network connectivity (not heterogeneity)
	output from centrality calculation
Exponent in Ψ: *m*	specific chemistry
	chosen bond weights
	free parameter from fit to moduli data
prefactor in Ψ: *exp*(*b*)	specific chemistry
	the representative network size chosen
	chosen bond weights
	free parameter from fit to moduli data

**Figure 3:**
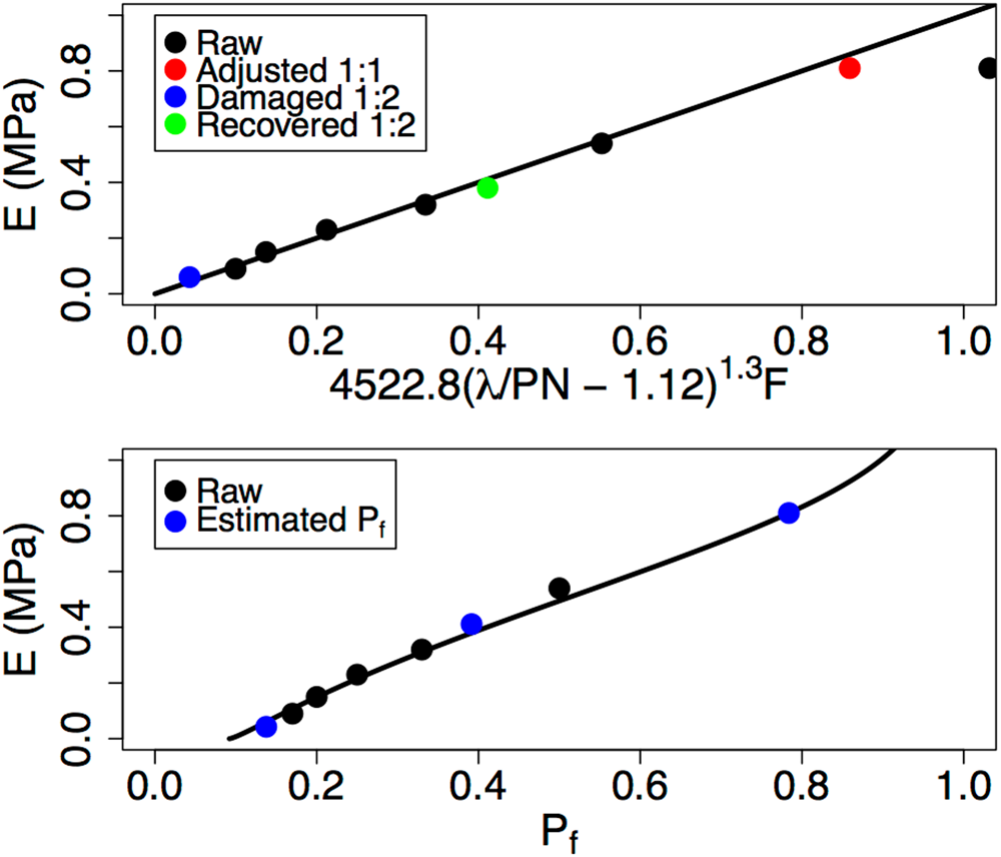
Data taken from Bao *et al*. The top plot contains the experimental modulus as a function of the two-parameter fit to the theory. The colored circles indicate where we invert the model to predict the value of the fraction of cross-linker bonds formed *P*_*f*_. The red circle is the outlier point adjusted with a more realistic value of *P*_*f*_. The blue is a damaged sample and the green is the same sample after one hour of recovery. The bottom plot shows the modulus as a function of *P*_*f*_ and the fitted theoretical curve.

Working within the model described above by Eq. 2 and the enumerated algorithm, we find that $E\approx 4522.8(\lambda /(PN)-{\rm{\Lambda}})^{1.3}F$ for this system when the five lowest concentrations of iron are considered. The value of Λ ≈ 1.12 is found by estimating *λ*/(*PN*) for the case of the probability of forming polymer-iron-polymer bonds *P*_*f*_ = 0.05. Figure 3 (top) plots Bao’s data in units of MPa in black circles as a function of this fit. The line is the ideal relationship. Note that the value excluded from the fit, that is, the black circle data point with the highest modulus value, falls well off the trend line. This data point corresponds to Bao’s 1:1 Fe:Hpdca-PDMS sample, a stoichiometry that should match one iron to two ligands (if the assumed efficiency described above is correct) and could, in principal, result in all ligands participating in polymer-iron-polymer bonds. Clearly, however, this is physically improbable, since kinetic effects will always prevent the complete formation of all of the possible bonds in the system. We may calculate the true fraction of bonds formed at this saturated concentration by inverting the relationship between the modulus and bonding probability *P*_*f*_. This leads to an estimate of *P*_*f*_ ≈ 79% for this iron concentration. Calculating an ensemble of adjacency matrices with that value produces the value along the horizontal axis for the red circle in Fig. 3 (top). Following the same procedure, we estimate the values of *P*_*f*_ for the case of the second highest iron concentration considered, the 1:2 Fe:Hpdca-PDMS sample, after damage induced by elongation to 1500% strain. The stress-strain curves immediately following the strain and after a one-hour recovery period were presented by Bao. From those curves, we estimate the corresponding values of the modulus. Given that data, we estimate that *P*_*f*_ ≈ 14% immediately after the 1500% strain and *P*_*f*_ ≈ 39% after one hour of recovery. Remarkably, given an original *P*_*f*_ = 50%, that translates into an 72% loss of iron cross-links after the damaging strain measurement with a subsequent recovery to a 22% loss after one hour. Experimental validation of our analysis and tracking of the recovered bond fractions as a function of time could provide interesting insight into the self-healing process. Figure 3 (bottom) presents all values of the modulus as a function of bonding probability, illustrating the overall dependence of the tensile properties on that parameter as captured by the model presented herein. Finally, we note that we employed an estimate of the efficiency of cross-linking by the FeCl_3_ (50%) in the analysis above. Experimental determination of this efficiency would improve the fidelity of our predictions for *P*_*f*_. We varied this value from 45% to 55% and found that both the exponent *m* and percolation threshold value Λ were insensitive to this choice. However, the prefactor *exp*(*b*) obtained values of 3816.6, 4522.8, and 3675.5 for efficiencies of 45%, 50%, and 55%, respectively. Similarly, *P*_*f*_ for the 1:1 Fe:Hpdca-PDMS sample varied as 74%, 79%, and 91% for those efficiencies. The same quantity for the 1:2 Fe:Hpdca-PDMS sample immediately following damage presented values of 12%, 14%, and 14%, while the recovered sample displayed values of *P*_*f*_ equal to 35%, 39%, and 45%. The analogous plots to Fig. 3 for those other cross-linking efficiencies are available in the Supplementary Information.

## Appendix

Below, we demonstrate analytically that only the value of the prefactor *exp*(*b*) depends on the choice of weights *w*_*k*_ in the adjacency matrix for the problem considered herein. In the calculations presented above, we use an ensemble average for both the partition function *Z* and the principal eigenvalue *λ*. In order for only the prefactor to depend on the choice of weights, we must both establish that the ensemble average 〈*Z*〉 is independent of that choice (since it dictates the value of the free energy term *F*) and find how Ψ varies with *w*_*k*_.

First, we show that $\langle \lambda \rangle \approx PN\sum {\alpha }_{k}{w}_{k}\equiv {\langle d\rangle }_{w}$. The expansion coefficients *α*_*k*_ are the fraction of formed bonds of type *k* (i.e. the fraction of all bonds formed that are cross-links, entanglements, or covalent bonds). The weights *w*_*k*_ are the corresponding weights for bond type *k*. The quantity 〈*d*〉_*w*_ is simply the weighted average degree of the ensemble of networks. Recall that the eigenvalue problem states that *A*_*i*,*j*_ *e*_*j*_ = *λe*_*i*_. Appealing to the approximations that $\langle {A}_{i,j}{e}_{j}\rangle \approx \langle {A}_{i,j}\rangle \langle {e}_{j}\rangle$, that $\langle \lambda {e}_{i}\rangle \approx \langle \lambda \rangle \langle {e}_{i}\rangle$, and that $\langle {e}_{j}\rangle \approx \langle {e}_{i}\rangle$ yields that the ensemble average adjacency matrix is $\langle {A}_{i,j}\rangle \approx \langle \lambda \rangle$. Note that the first two of these approximations relies on the recognition that the adjacency matrix, the principal eigenvector, and the principal eigenvalue are random variables that may be approximated as independently distributed. On the other hand, we may expect that $\langle {A}_{i,j}\rangle \approx {\langle d\rangle }_{w}\equiv PN\sum {\alpha }_{k}{w}_{k}$ since each node except the diagonal terms in the adjacency matrix must take on the average value of the weighted degree. The diagonal itself is, of course, filled with zeros. Thus, we may write that $\langle \lambda \rangle /PN\approx \sum {\alpha }_{k}{w}_{k}$. We find this expression to faithfully predict the numerical estimates of 〈*λ*〉/*PN* and will use it below.

Next, let us establish that the ensemble average principal eigenvector does not change with a transformation of the weights. Consider two sets of weights *w*_*k*_ and ${w}_{k}^{^{\prime} }$. The ratio of the two values of 〈*λ*〉/*PN* recovered for a given value of *P* is a constant determined by the two choices of weights and is given by

4 $$\frac{\langle \lambda \rangle /PN}{\langle \lambda ^{\prime} \rangle /PN}=\frac{\langle \lambda \rangle }{\langle \lambda ^{\prime} \rangle }=\frac{\sum {\alpha }_{k}{w}_{k}}{\sum {\alpha }_{k}{w}_{k}^{^{\prime} }}\equiv R.$$

Note that the values of *α*_*k*_ and *PN* do not change if we change the weights.

Thus, we may write that $\langle \hat{A}\rangle \approx R\langle \hat{A}^{\prime} \rangle$ and $\langle \hat{A}\rangle \langle \vec{e}^{\,\prime} \rangle \approx R\langle \lambda ^{\prime} \rangle \langle \vec{e}^{\,\prime}\rangle$. That is, only the eigenvalue changed with the transformation of the weights. We find this to be in excellent agreement with our numerical evaluation of the free energy *F* under changes in the choices of weights.

Finally, we probe how Ψ varies under a change of weights for the particular problem addressed herein. The two models must be roughly equivalent, so we have that Ψ = Ψ′. Therefore,

5 $$exp(b^{\prime} ){[\frac{\lambda ^{\prime} }{PN}-{\rm{\Lambda }}^{\prime} ]}^{m^{\prime} }=exp(b){[\frac{\lambda }{PN}-{\rm{\Lambda }}]}^{m}.$$

Using the expansion above for the *λ*/*PN*, noting that the same expansion must hold for Λ, and introducing the fraction of each bond type at the percolation threshold *β*_*k*_, we may write

6 $$exp(b^{\prime} ){[\sum {\alpha }_{k}{w}_{k}^{^{\prime} }-\sum {\beta }_{k}{w}_{k}^{^{\prime} }]}^{m^{\prime} }=exp(b){[\sum {\alpha }_{k}{w}_{k}-\sum {\beta }_{k}{w}_{k}]}^{m}.$$

Now, group the factors in terms of *w*_*k*_ and ${w}_{k}^{^{\prime} }$ by defining ${{\rm{\Delta }}}_{k}\equiv {\alpha }_{k}-{\beta }_{k}$ to obtain

7 $$exp(b^{\prime} ){[\sum {{\rm{\Delta }}}_{k}{w}_{k}^{^{\prime} }]}^{m^{\prime} }=exp(b){[\sum {{\rm{\Delta }}}_{k}{w}_{k}]}^{m}.$$

For the specific scenario considered herein, only one type of bond changes in number as we move from the percolation threshold to the condition set by adding a given amount of FeCl_3_, the polymer-iron-polymer cross-links. Thus,the values for Δ_*k*_ are zero for the entanglements and the covalent bonds; only one term of Δ_*k*_ survives in the sum, that associated with the cross-links. We may therefore write

8 $$exp(b^{\prime} ){[w^{\prime} {\rm{\Delta }}]}^{m^{\prime} }=exp(b){[\sum w{\rm{\Delta }}]}^{m}$$

where the surviving terms of Δ, *w*, and *w*′ correspond to the cross-links. Therefore, we see that changing the weights only impacts the value of *b* and *m*, in general. If we fix $m\equiv m^{\prime}$, we may write a simple relationship for the change in *b* under the change of weights. For the particular system studied herein, we find that

9 $$b^{\prime} -b=m\,\mathrm{log}(w/w^{\prime} ).$$

## Electronic supplementary material

Supplementary info
