# Supplementary material for: Eigenvector Centrality is a Metric of Elastomer Modulus, Heterogeneity, and Damage
Source: Sci Rep. 2017 Apr 27;7:1241. doi: 10.1038/s41598-017-00818-0 (PMC5430702; doi:10.1038/s41598-017-00818-0)
Supplement: Supplementary file 1 — Supplementary info [file 41598_2017_818_MOESM1_ESM.pdf]

Eigenvector Centrality is a Metric of Elastomer Modulus, Heterogeneity, and Damage

P. M. Welch[1,*] **and C. F. Welch**[2]

[1]Theoretical Division, Los Alamos National Laboratory, Los Alamos, New Mexico 87544
[2]Materials Science and Technology Division, Los Alamos National Laboratory, Los Alamos, New Mexico 87544
[*]PWelch@lanl.gov

ABSTRACT

Here, we provide additional plots for the model of the Young's modulus with two different assumptions for the cross-linker efficiency.

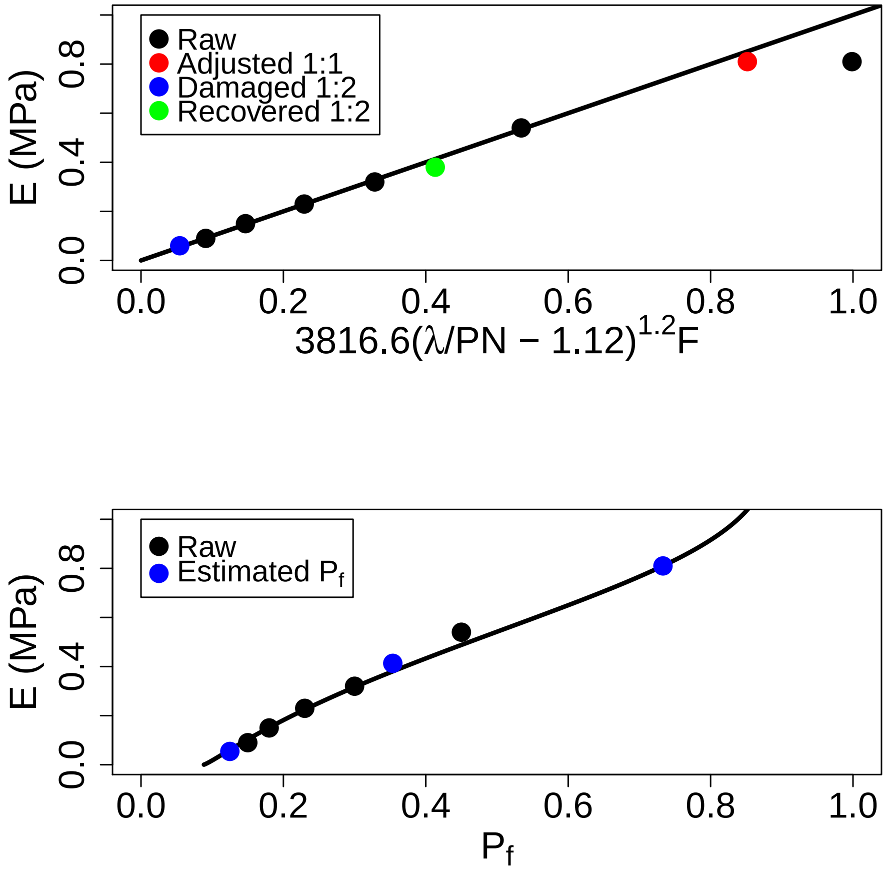

Figure S1: Data taken from Bao *et al*. The top plot contains the experimental modulus as a function of the two-parameter fit to the theory. The colored circles indicate where we invert the model to predict the value of the fraction of cross-linker bonds formed P_f. The red circle is the outlier point adjusted with a more realistic value of P_f. The blue is a damaged sample and the green is the same sample after one hour of recovery. The bottom plot shows the modulus as a function of P_f and the fitted theoretical curve. Here, we have assumed a 45% efficiency for the $FeCl_3$ cross-linker.

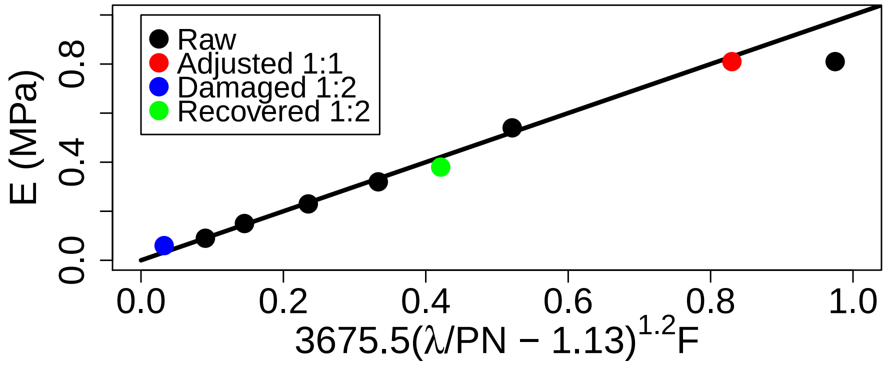

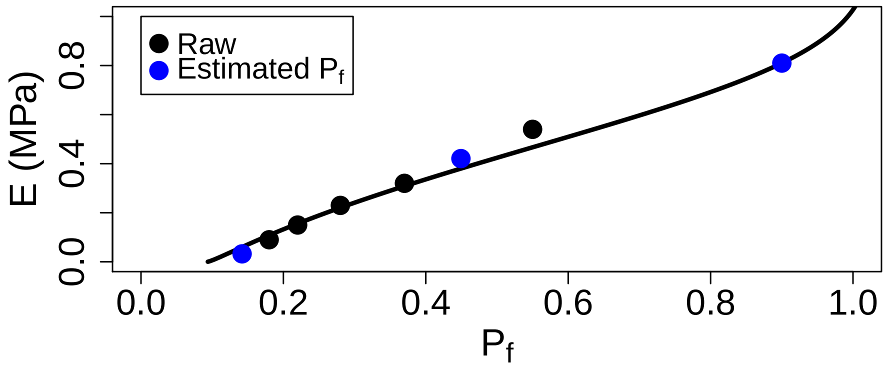

Figure S2: Data taken from Bao *et al*. The top plot contains the experimental modulus as a function of the two-parameter fit to the theory. The colored circles indicate where we invert the model to predict the value of the fraction of cross-linker bonds formed P_f. The red circle is the outlier point adjusted with a more realistic value of P_f. The blue is a damaged sample and the green is the same sample after one hour of recovery. The bottom plot shows the modulus as a function of P_f and the fitted theoretical curve. Here, we have assumed a 55% efficiency for the $FeCl_3$ cross-linker.
